# Poster Session I- A22 INVESTIGATING THE ROLE OF YAP/TAZ IN GASTRIC METAPLASIA AND CANCER DEVELOPMENT

**DOI:** 10.1093/jcag/gwaf042.022

**Published:** 2026-02-13

**Authors:** J Sung, A Ju, S Gruenheid, A Gregorieff

**Affiliations:** Microbiology and Immunology, McGill University, Montreal, QC, Canada; Microbiology and Immunology, McGill University, Montreal, QC, Canada; Microbiology and Immunology, McGill University, Montreal, QC, Canada; Research Institute of the McGill University Health Centre, Montreal, QC, Canada

## Abstract

**Background:**

Gastric cancer is the third deadliest cancer, often arising from chronic gastritis caused by *Helicobacter pylori*, which can lead to ulcers and excessive cell growth that may trigger tumor formation. This transition to gastric cancer is closely associated with metaplastic cell lineages in the epithelium, notably the appearance of proliferative mucus-producing cells at the base of the gastric glands, known as spasmolytic polypeptide/trefoil factor 2-expressing metaplasia (SPEM). Despite this understanding, the processes driving alterations in cell fate during chronic inflammation and their function in regeneration and tumorigenesis are poorly understood. A critical determinant of cell fate regulation in regenerating tissues is the Hippo pathway. Using an acute chemical injury model to initiate SPEM, based on administration of high-dose tamoxifen (HDT), our findings demonstrated that inactivation of the downstream Hippo effectors, Yap and Taz, in the murine gastric epithelium impaired SPEM resolution and triggered chronic gastritis.

**Aims:**

Thus, **we hypothesize** that the transcriptional activators Yap/Taz play an essential role in regulating epithelial-immune crosstalk during metaplasia and gastric cancer development. To address this hypothesis, we aim to dissect the direct immunomodulatory roles of these effectors by identifying epithelial-specific Yap/Taz responsive genes and functionally characterizing Yap/Taz-dependent immune responses. We also seek to investigate how Yap/Taz regulate epithelial cell fate during tissue repair, where their dysfunction may impair epithelial healing, further promote inflammation, and contribute to cancer development.

**Methods:**

Yap/Taz mutant mice were transcriptionally profiled to identify epithelial specific immunomodulatory signals as well as to uncover alterations in tissue regeneration pathways. Immune cell populations in the lamina propria of Yap/Taz-deficient mice were characterized by flow cytometry, and their functional roles during SPEM were further investigated using *in vivo* ablation experiments. Using a genetic cancer model with additional knockout of the tumor suppressor p53, we investigated whether the observed roles of Yap/Taz are implicated within a tumorigenic context.

**Results:**

Our data revealed that Yap/Taz-deficient epithelium exhibits altered regulation in pathways related to chemotaxis and barrier integrity, which may underlie the associated inflammation. Further immune profiling demonstrated that loss of Yap/Taz resulted in chronic B cell and macrophage infiltration in corpus glands, supporting an immunomodulatory role for these effectors.

**Conclusions:**

Through this project, we aim to define biological and immunological responses that are controlled by Yap/Taz, providing important insights on immune-epithelial cell interactions important for tissue regeneration and tumorigenesis.

**Funding Agencies:**

CIHRFRQS

